# 

**Published:** 1907-02

**Authors:** 


The Therapeutics of Laughter.

VOL. XXII. FEBRUARY, 1907. No. 8.

[illegible]
SURGEON GENERAL'S OFFICE
MAR.-1--1907

### TEXAS MEDICAL JOURNAL

"RED BACK"

PUBLISHED AT AUSTIN, TEXAS, BY
F. E. DANIEL, M. D., - - Proprietor
PRINTED BY THE VON BOECKMANN-JONES CO.

Subscription, $1.00 a Year, in Advance. - - - Single Copies, 10 Cents

Entered at the Postoffice at Austin, Texas, as Second-class Mail Matter

